# The importance of simulation education for the management of traumatic cardiac injuries: a case series

**DOI:** 10.1186/s40792-019-0762-y

**Published:** 2019-12-20

**Authors:** Takashi Nagata, Tomohiko Akahoshi, Michiko Sugino, Wataru Ishii, Ryoji Iizuka, Takafumi Shinjo, Yoshimitsu Izawa, Michiaki Hata, Alan Kawarai Lefor

**Affiliations:** 1 Department of Advanced Medical Initiatives, Division of Disaster and Emergency Medicine, Faculty of Medical Sciences, Kyushu University, Fukuoka, Japan; 2 Department of Emergency and Critical Care, Kyushu University Hospital, Fukuoka, Japan; 3 Department of Emergency and Critical Care, Kyoto 2nd Red Cross Hospital, Kyoto, Japan; 4 Department of Emergency and Critical Care, Jichi Medical University, Shimotsuke, Japan; 5 Department of Surgery, Yonemori Hospital, Kagoshima, Japan; 6 Department of Surgery, Jichi Medical University, Shimotsuke, Tochigi, Japan

**Keywords:** Penetrating cardiac injury, Left anterior thoracotomy, Blunt cardiac rupture, Median sternotomy

## Abstract

**Background:**

The management of cardiac trauma requires rapid intervention in the emergency room, facilitated by a surgeon with prior experience to have good outcomes. Many surgeons have little experience in the requisite procedures. We report here 4 patients who suffered cardiac trauma, and all 4 patients survived with good neurologic outcomes.

**Case presentations:**

Patient 1 suffered blunt cardiac trauma from a motor vehicle accident and presented in shock. Cardiac tamponade was diagnosed and a cardiac rupture repaired with staples through a median sternotomy after rapid transport to the operating room. Patient 2 suffered blunt cardiac trauma and presented in shock with cardiac tamponade. Operating room median sternotomy allowed extraction of pericardial clot with recovery of physiologic stability. Patient 3 presented with self-inflicted stab wounds to the chest and was unstable. She was brought to the operating room and thoracotomy allowed identification of a left ventricle wound which was repaired with a suture. Patient 4 presented in cardiac arrest with multiple self-inflicted stab wounds to the chest. Emergency room thoracotomy allowed repair of a right ventricle laceration with recovery of vital signs.

**Conclusions:**

The management of all 4 patients was according to the principles taught in the ATOM course. Three of the 4 surgeons had no prior experience with management of cardiac trauma and credited the good outcomes to taking the ATOM course. These are uncommon injuries and formal training in their management is beneficial to patients.

## Background

Traumatic cardiac injuries are rare and are usually associated with a high mortality rate. To save a patient with a blunt or penetrating cardiac injury, rapid diagnosis, decisions, and treatment by an experienced surgeon is mandatory [1]. The Advanced Trauma Operative Management (ATOM) course, which is a program of the American College of Surgeons and administered in Japan through the Japan Surgical Society, is a 1-day trauma course that includes a series of lectures and live-animal surgery simulation training with pigs to teach the surgical management of penetrating traumatic injuries [2]. The ATOM course includes a session about the management of cardiac injuries [3]. In Japan, the ATOM course started in 2008. Currently, there are six training sites (Hokkaido University Hospital, Tohoku University Hospital, Jichi Medical School, Teikyo University Hospital, Osaka City University Hospital, and Kyushu University Hospital), more than 50 instructors, and about 300 providers. It is difficult to confirm the effectiveness of the ATOM course introduction in Japan.

We present here four patients treated at four different centers in Japan who suffered traumatic cardiac injuries and were treated by four surgeons who took the ATOM course in Japan. The effectiveness of learning surgical skills by simulation education in a course such as ATOM is considered.

## Case presentations

Ethical approval was obtained for the presentation of each patient from each treating hospital. All patients consented to the publication of their information as shown.

### Patient 1

**Fig. 1:**
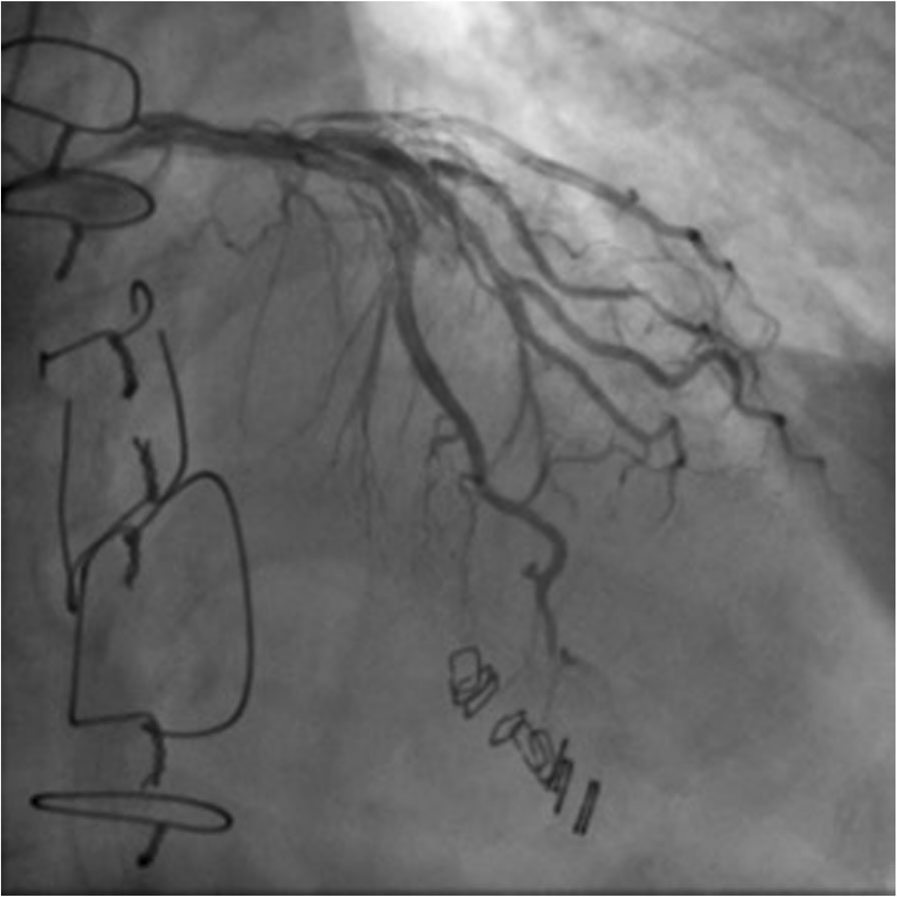
Postoperative imaging shows staples in place after repair of a penetrating cardiac injury with patent coronary arteries (patient 1)

A 67-year-old male bicycle rider was injured in a motor vehicle accident and brought to the emergency room (Glasgow Coma Scale (GCS) 7 (E1V2M4)), blood pressure (BP) 138/46 mmHg, respiratory rate (RR) 20/min, pulse (P) 80/min). Evaluation showed pericardial tamponade on Focused Assessment by Sonography for Trauma (FAST) ultrasound examination. The pericardium was drained with a needle and a pericardial window performed in the emergency room. This released the tamponade in less than 5 min, but the patient became hemodynamically unstable. The patient was rapidly transported to the operating room and median sternotomy performed. A right ventricle anterior wall rupture found and initially controlled with digital pressure and then repaired using a skin stapler, as taught in the ATOM course, then reinforced by *pledgeted sutures and the chest irrigated and closed.* Figure 1 shows the coronary angiography performed 37 days after the injury. The patient recovered without neurological deficits and was discharged home 50 days postoperatively.

### Patient 2

A 20-year-old female automobile driver was injured in a collision with a truck. She presented alert and oriented *(GCS15 (E4V5M6))* with hypotension (BP 100/70 mmHg, P 80/min, *SpO*_*2*_ 100% (on 100% O2 by face mask). Evaluation revealed cardiac tamponade, left lung contusion, liver injury, and right femoral shaft fracture. The patient remained stable was transferred to the operating room. After median sternotomy and incision of the pericardium (total time 1 h after admission), 60g of clot was found. By removing this clot, compression was released. No heart injury was found. The injuries of the left lung and liver required no specific treatment, and the right femur was repaired by external fixation by an orthopedic surgeon following the cardiac procedure. She was discharged 80 days postoperatively without complications.

### Patient 3

**Fig. 2:**
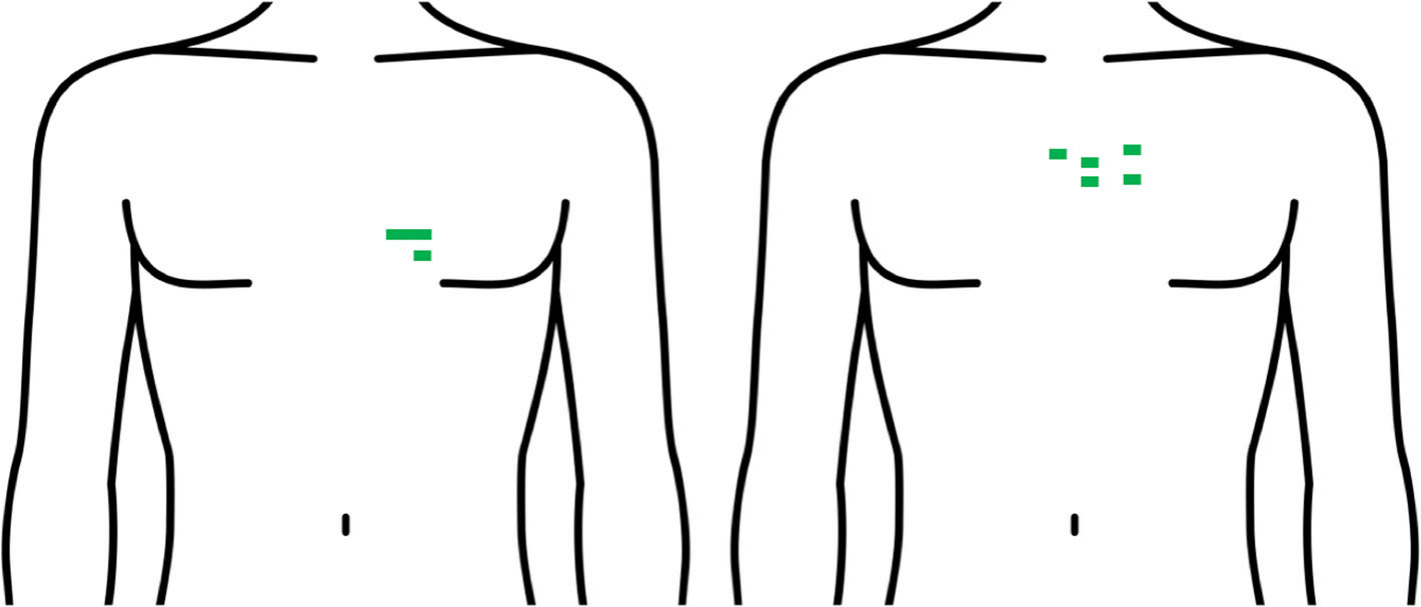
Location of stab wounds in patients 3 (left panel) and 4 (right panel). The green lines indicate the location of the stab wounds. (The figure was obtained from https://pixta.jp/illustration/37387380 and is reproduced with permission)

A 32-year-old female attempted suicide with *two self-inflicted stab wounds* to the left chest lateral to the sternum at the level of the seventh rib (Fig. 2, left). She presented to the ER with hypotension (BP 100/60 mm Hg, *P 80/min*, RR 20/min, GCS 15 on arrival), rapidly deteriorated, and left anterior thoracotomy was immediately performed in the operating room. A left ventricular laceration and a left upper lobe laceration were identified, with no evidence of cardiac tamponade. The left ventricle stab wound was closed immediately with a single polypropylene suture with a pledget, the left interthoracic artery ligated, and a partial pulmonary lobectomy done. The patient was discharged from the hospital 14 days after injury.

### Patient 4

A 46-year-old male suffered multiple self-inflicted stab wounds to the left chest from a knife. The wounds were 2-cm long with the deepest wounds located just lateral to the sternal border at the level of the third rib (Fig. 2, right). During transport, this patient was in shock and had a complete cardiopulmonary arrest upon arrival. Emergency room left anterior thoracotomy was performed, and rupture of the right ventricular wall identified with evidence of cardiac tamponade. The epicardium was released within 14 min of presenting to the Emergency Room. This was repaired with a polypropylene suture with a pledget. *The patient suffered another cardiopulmonary arrest supported with 22 min of cardiopulmonary resuscitation after which he regained spontaneous cardiac function*. The blood pressure was labile, and the patient was transferred to the operating room where extracorporeal membrane oxygenation (VA-ECMO) was initiated, and the patient transferred to the intensive care unit. Five days after injury, patient was withdrawn from ECMO, and 8 days after injury, he was extubated and discharged from the intensive care unit without neurological complications. This patient has previously been reported in part [4]. The patient was discharged from the hospital 13 days after injury.

## Discussion

The prognosis of patients after traumatic cardiac injuries is poor in general, especially those who present in cardiac arrest. The management and outcomes of penetrating and blunt cardiac injuries are somewhat different. The National Trauma Data Bank (United States) reported that the percentage of the patients transferred to hospital with blunt cardiac rupture among all patients with blunt traumatic injuries is just 0.045% [5]. The mortality rate of patients after blunt cardiac injuries is especially high, and almost all patients with blunt cardiac injuries are dead at the scene. The mortality rate of patients after blunt traumatic cardiac injuries who present to the emergency room with vital signs is 89% [5].

We present four patients here with different mechanisms of cardiac injury including two after blunt injuries and two after penetrating injuries that represent a wide range of cardiac injuries (summarized in Table 1). These injuries are extremely rare in Japan, and most surgeons have never seen a patient with these injuries. Thus, few surgeons have prior experience which is so important for the prompt evaluation and management of these injuries.

**Table 1:** Clinical characteristics of 4 patients who survived traumatic cardiac injuries

Patient	Age, years	M/F	Mechanism	External injury	Clinical condition in ER	Treatment	Discharge, days
1	67	M	Blunt	–	BP 138/46, GCS7, tamponade	*ER pericardial drainage, then* OR sternotomy, stapling	50
2	20	F	Blunt	Femur fracture	BP100/70, tamponade	OR sternotomy, extract clot	80
3	32	F	Stab, 1 wound	Left chest at 7th rib	BP100/60, no tamponade	ER left anterior thoracotomy, suture left ventricle	14
4	46	M	Stab, 2 wounds	Left chest at 3rd rib	Cardiac arrest, tamponade	ER left anterior thoracotomy VA-ECMO	13

*M* male, *F* female, *ER* emergency room, *VA-ECMO* venous-arterial extracorporeal membrane oxygenation, *OR* operating room, *GCS* Glasgow Coma Scale

In Japan, the trauma training course for physicians in the emergency department, is the Japan Advanced Trauma Evaluation and Care (JATEC), which is widely conducted. In JATEC, the diagnosis and treatment for cardiac tamponade is taught as detecting by focused assessment sonography on trauma, and pericardial puncture. To save a patient with a cardiac injury, surgical repair is essential, and the ATOM course could contribute to teaching a standard approach to the care of these injuries.

The initial evaluation of the patients reported here was performed according to JATEC and includes airway, breathing, circulation, dysfunction of CNS, and exposure and environmental control. A FAST scan is rapidly performed to evaluate the pericardium. The importance of ultrasound in the evaluation of patients following cardiac trauma is well known and may even include extended examinations in a stable patient [6, 7]. The literature suggests that FAST scan has a sensitivity of approximately 97% and a specificity of 100% [8]. While some have suggested that CT scan may be useful in the evaluation of cardiac injuries, the specificity and sensitivity are far below that of FAST scan and it was concluded that CT scan should not be used [8].

The role of Emergency Room thoracotomy has been discussed in the management of cardiac traumatic injuries [9]. In a meta-analysis of emergency room thoracotomy, more were performed in patients with penetrating injuries than blunt injuries [10]. Survival in the penetrating injury group was a mean of 17% (2.7–37.5%), and for blunt injuries was a mean of 4.6% (0.6–60%). Among patients with penetrating injuries, mean percentage of neurologically intact survivors was 86% and only 12% in the patients with blunt injuries.

There are some very subtle differences among penetrating injuries to the heart which must be considered in the overall management of these injuries [11]. The cause of the injury dictates the ideal management [12]. Small wounds with tamponade may be successfully treated only with pericardiocentesis. This may be unsuccessful especially when there is a large coagulum in the pericardial space, and in those cases, surgical intervention (pericardial window or thoracotomy) may be necessary. Larger wounds clearly need emergent thoracotomy and cardiorrhaphy. It is estimated that nearly 90% of patients with penetrating cardiac injuries never reach the hospital [12]. Blunt cardiac injuries require a high index of suspicion to establish the diagnosis [13].

Once a cardiac injury is found, direct exploration of the injury is advocated [6]. Due to its anatomical location, injuries to the right ventricle are most common. The ATOM course teaches the use of the skin stapler to temporize and stop the bleeding followed by the placement of sutures with pledgets into the myocardium, as was performed in patients 1, 3, and 4 in this report [3]. Cardiac stapling has also been reported elsewhere [14].

Other approaches have also been tried. In patient 4 in this series, ECMO was used as an adjunct. The use of ECMO to support a patient after a traumatic injury has been reported only one time before [15].

In a series of 60 patients who sustained penetrating cardiac injuries, the overall survival was 37% (22/60) including 5/35 patients with gunshot wounds and 17/25 patients with stab wounds [16]. ERT was performed in 37/60 patients, with 6/37 survivors (16%). Patients without vital signs on arrival had a mortality of 96%. Factors which predicted outcomes were mechanism of injury and the presence of sinus rhythm upon opening the pericardium.

These four patients survived because of prompt and aggressive action by surgeons who took the ATOM course in Japan. Only one of the four surgeons involved in the care of these patients had ever treated a cardiac injury. The experience for three of the four surgeons was limited to the ATOM course. The outcomes of these patients were uniformly excellent; all patients recovered without neurologic sequelae. This is particularly rare in patients after blunt cardiac injuries as highlighted by patient 1 in this series who had a ventricular rupture after a blunt injury, as well as patient 4 who had a complete cardiac arrest at the time of presentation. The care of trauma patients requires a team approach for the entire spectrum of care. The ATOM course emphasizes the importance of the team through an education program that includes specific training for nurses as members of the trauma team.

This report is notable in several aspects. First, we present four patients with traumatic cardiac injuries who survived without neurologic sequelae. This is only the second case report in the literature of a patient treated for a cardiac injury with ECMO. Finally, three of the four surgeons only experience with surgical management of a cardiac injury was in an animal simulation laboratory.

## Conclusion

The ATOM course provided these surgeons with experience in basic techniques in the management of cardiac injuries. While it is not possible in a simulation course such as ATOM to provide experience with every possible clinical situation, the course gave the participants confidence when they approached injuries they had not previously encountered. Since cardiac injuries are rare in Japan, there was no prior formal training available in the necessary procedures. The effectiveness of the ATOM course and simulation training to learn procedures for which clinical experience is rare, is suggested by these four patients. Efforts should be continued for simulation training for trauma care in Japan.

## Data Availability

The data supporting the conclusions of this article are included within the article.

## Abbreviations

ATOM: Advanced Trauma Operative Management
BP: Blood pressure
CT: Computed tomography
ECMO: Extracorporeal membrane oxygenation
ERT: Emergency room thoracotomy
FAST: Focused Assessment by Sonography for Trauma
GCS: Glasgow Coma Scale
JATEC: Japan Advanced Trauma Evaluation and Care
P: pulse
RR: Respiratory rate

## References

[1] Gosavi Sucheta, Tyroch Alan H., Mukherjee Debabrata (2016). Cardiac Trauma. Angiology.

[2] Lefor Alan Kawarai (2018). Trauma surgery simulation education in Japan: the Advanced Trauma Operative Management course. Acute Medicine & Surgery.

[3] Jacobs LM, Luk SS (2010). The cardiovascular system. Advanced Trauma Operative Management, 2nd Edition.

[4] Sugino M, Tanaka Y, Nagao Y (2017). A case of stabbing cardiac rupture saved by preparing with emergency room thoracotomy. J Japanese Assoc Acute Med.

[5] Teixeira PGR, Inaba K, Oncel D (2009). Blunt cardiac rupture: a 5-year NTDB analysis. J Trauma.

[6] Hsu Hung-Lung, Chen Jer-Shen (2015). Penetrating cardiac injury: Consider direct exploration and “finger haemostasis”, and remember to screen for intra-cardiac injury after a successful repair. Injury.

[7] Saranteas Theodosios, Mavrogenis Andreas F., Mandila Christina, Poularas John, Panou Fotios (2017). Ultrasound in cardiac trauma. Journal of Critical Care.

[8] Góes Junior AMO, Oliveira ÉVL, Albuquerque FBA, Martins EG, Andrade MC, Abib SCV (2019). The use of computed tomography for penetrating heart injury screening. Rev Col Bras Cir..

[9] Fairfax Lindsay M., Hsee Li, Civil Ian D. (2014). Resuscitative Thoracotomy in Penetrating Trauma. World Journal of Surgery.

[10] Tan BK, Pothiawala S, Ong ME. Emergency thoracotomy: a review of its role in severe chest trauma. Minerva Chir. 2013;68(3):241-250. PMID: 23774089 23774089

[11] Asensio Juan A., Stewart B. Montgomery, Murray James, Fox Arthur H., Falabella Andres, Gomez Hugo, Ortega Adrian, Fuller Clark B., Kerstein Morris D. (1996). PENETRATING CARDIAC INJURIES. Surgical Clinics of North America.

[12] Lateef Wani Mohd, Gani Ahangar Ab, Nabi Wani Shadab, Irshad Ifat, Ul-Hassan Nayeem (2012). Penetrating Cardiac Injury: A Review. Trauma Monthly.

[13] Bellister Seth A., Dennis Bradley M., Guillamondegui Oscar D. (2017). Blunt and Penetrating Cardiac Trauma. Surgical Clinics of North America.

[14] Macho JR, Markison RE, Schecter WP (1993). Cardiac stapling in the management of penetrating injuries of the heart: rapid control of hemorrhage and decreased risk of personal contamination. J Trauma..

[15] Gatti Giuseppe, Forti Gabriella, Bologna Alessandro, Sagrati Gianfranco, Gustin Gianfranco, Korcova Renata, Benci Elisabetta, Visintin Luca (2014). Rescue extracorporeal membrane oxygenation in a young man with a stab wound in the chest. Injury.

[16] Asensio J (1998). Penetrating Cardiac Injuries: A Prospective Study of Variables Predicting Outcomes. Journal of the American College of Surgeons.

